# Emulgels Structured with Dietary Fiber for Food Uses: A Rheological Model

**DOI:** 10.3390/foods11233866

**Published:** 2022-11-30

**Authors:** Elisabetta Bruno, Francesca Romana Lupi, Domenico Mammolenti, Olga Mileti, Noemi Baldino, Domenico Gabriele

**Affiliations:** Department of Information, Modeling, Electronics and System Engineering, (D.I.M.E.S.), University of Calabria, Via P. Bucci, Cubo 39C, 87036 Rende, Italy

**Keywords:** emulgels, dietary fiber, particle gels, suspensions, rheological modelling, microstructure

## Abstract

Emulgels are biphasic emulsified systems in which the continuous phase is structured with a specific gelling agent. In this work, a rheological and microscopic investigation of O/W emulgels prepared by structuring the aqueous (continuous) phase with citrus fiber was carried out with the aim of designing their macroscopic properties for food uses and predicting their characteristics with a rheological model. According to previous investigations, fiber suspensions behave as “particle gels” and, consequently, the derived emulgels’ properties are strongly dependent on the fiber concentration and on process conditions adopted to produce them. Therefore, a rotor–stator system was used to prepare emulgels with increasing fiber content and with different levels of energy and power used for mixing delivered to the materials. An investigation of particle gels was then carried out, fixing the operating process conditions according to emulgel results. Furthermore, the effect of the dispersed (oil) phase volume fraction was varied and a modified semi-empirical Palierne model was proposed with the aim of optimizing a correlation between rheological properties and formulation parameters, fixing the process conditions.

## 1. Introduction

Emulsion gels or emulgels are biphasic systems formed by dispersing a viscous unstructured phase in a structured continuous phase [1,2]. The structuration of the continuous phase can reduce the movement of the dispersed phase, increasing its stability [3,4].

Emulgels are widely used in pharmaceutical and cosmetic preparations as controlled-release materials [5,6,7] and, in the case of dermatological use, they have several favorable properties such as spreadability, removability, emollience and water solubility (in the case of O/W systems) [8]. 

The main properties of emulgels (such as spreadability, texture, control of delivery rate of active components, etc.) are strictly related to rheological characteristics and, in turn, to microstructure; therefore, the proper design of emulgels, having the desired sensorial and functional properties, needs the knowledge and control of their rheological characteristics [9,10].

Regarding this, the rheological, mechanical and textural properties can be tuned by varying the properties of the structured phase and the volumetric ratio between phases; this also makes these systems interesting and versatile for food uses where different types of emulsions are widely used [1]. In the last few years, emulsion gels have been used as fat replacers in the production of functional foods. Chen et al. [11] investigated the effect of emulgels enriched with β-carotene as a margarine alternative for sponge cakes to decrease the total saturated or trans-fat content in bakery products. Nasirpour-Tabrizi et al. [12] studied the rheological and physicochemical properties of low-fat spreadable emulgels containing ω-3 fatty acids in a matrix of carrageenan, xanthan gum and maltodextrin. Oppong et al. [13] proposed rice flour-based emulgels for producing healthier oven-baked fish nuggets; according to the authors, this technology allows the lipid content to be controlled and lipid oxidation to be delayed. 

Different gelling agents were proposed to structure the water phase (that is, the usual dispersing medium) [2], such as polysaccharides [11,12,13], proteins [14,15,16], mixtures of them [17] and, more recently, dietary fiber [18,19] for functional food applications.

Dietary fiber has a positive effect on the everyday diet; regular consumption can reduce the risk of developing certain pathologies such as hypercholesterolemia, diabetes or gastro-intestinal disorders [20,21,22,23]. Presently, owing to the increase in consumption of ready-to-eat foods, the intake of dietary fiber in the daily diet is below the recommended levels; the World Health Organization has suggested a reduction in fat content in the diet, and an increase in the consumption of dietary fiber [24]. For these reasons, the food industry started to investigate new products characterized by textural properties comparable to those of commercial ones, but with a lower content of fats and calories and, if possible, an increased fiber content. It is worthy to say that, in addition to nutritional properties, dietary fibers have important functional characteristics such as gelling and texturizing properties, stabilizing effects, water-binding capacity and, consequently, an increased shelf life in food products [25,26,27]. Owing to its properties, dietary fiber can help in designing low-fat dressing or mayonnaise, generally regarded as high-caloric and high-fat foods because of the quantity of oil and eggs used in their recipes. The presence of fiber is helpful in tuning the adequate rheological properties of these dressings, decreasing the fat content.

A number of relevant papers investigated the rheological or mechanical properties of emulgels (see, for instance, the review by Geremias-Andrade et al. [2]) and many of them discussed the modelling of rheological properties and their relationship to microstructure and formulation (see, for instance, [7,14,15,16,28,29,30,31,32]). In many of these works, fillers stiffer than the matrix are observed yielding an increase in the emulgel modulus with respect to that of dispersing phase. Moreover, owing to the characteristics of the two phases, few works [15,28,31,32] take into account the potential effects of interfacial properties (such as the interfacial tension) between dispersed and continuous phases. Nevertheless, when a liquid oil is used within a viscoelastic matrix, the ratio between filler and matrix shear moduli is lower than unity and a reduction in emulgel moduli with respect to the unmodified gel can be observed [7,33] yielding to the need of potential modifications of commonly adopted models. 

In the present work, different from other works in the literature, sunflower oil emulgels structured with dietary fiber and characterized by a filler less consistent than the matrix were investigated with small-amplitude oscillations, and the complex modulus was related to composition and quantity of single phases, trying to also take into account the potential contribution of the interface between oil and water phases. In the experimental part of this paper, the effects of fiber concentration and mixing conditions on the rheological properties of emulgels were investigated, as a preliminary step to determine the proper operating conditions (i.e., mixing speed and time) for sample preparation. Afterwards, the oil volume fraction was also varied and the rheological properties of emulgels were studied and modelled.

## 2. Materials and Methods

Materials used to prepare emulgels were distilled water, commercial sunflower oil (De Santis, Italy), soybean lecithin powder (Lecico GmbH, Germany) as an emulsifier and dietary citrus fiber (kindly supplied by JRS Silvateam Ingredients Srl, Italy). 

### 2.1. Preparation of Single Phases

The oil phase was prepared by adding the lecithin powder (15% w/w) to the continuously stirred sunflower oil at 70 °C with a magnetic heater-mixer until the complete melting of the powder (about 30 min). 

The aqueous phase (fiber suspension) was prepared with an Ultra Turrax (UT) T50 (Ika-Werke GmbH, Germany) rotor–stator device equipped with a G45F tool adding amounts of fiber to water at room temperature and varying, for each sample, the fiber concentration in the suspension while keeping the operating conditions constant (i.e., mixing speed and time) in order to have a specific energy (*E_s_*) equal to 45.2 J/g and a specific power (*P_s_*) equal to 0.151 W/g (corresponding to 8000 rpm for 300 s).

The specific power of mixing of the UT T50 was evaluated with the calorimetric determination described by Bruno et al. [34] as a function of the mixing speed Ω (in rpm); the resulting relation between *P_s_* (in W) and Ω is:

(1) $$P_{s}=1.59\cdot 10^{-10}\Omega^{2.3}$$

Therefore, the specific energy *E_s_* (J/g) can be calculated as:

(2) $$E_{s}=P_{s}\cdot t$$

where *t* is the time of mixing (in seconds). 

The adopted conditions were chosen according to the results obtained in a previous work [34], where it was observed that they correspond to a full dispersion of the fiber in water and, therefore, to an asymptotic trend of rheological properties that become independent of the adopted dispersing conditions. It is worth noticing that, even though operating conditions adopted in this work are similar to those used in the previous study [34], different values of rheological parameters were found because different types of citrus fiber were used. Investigated samples are listed in Table 1. 

### 2.2. Preparation of Emulgels 

Emulgels (100 g batches) were prepared by dispersing the oil phase in the aqueous phase using the same Ultra Turrax (UT) T50 (Ika-Werke GmbH, Germany) rotor–stator device used for fiber suspensions. The oil phase (cooled down to room temperature) was slowly added to the aqueous phase. The samples were homogenized at different mixing speeds W corresponding to different specific powers of mixing *P_s_*, as reported in Table 2. The dispersion time used was chosen by aiming at guaranteeing a fixed value of mixing energy for all samples. 

Emulgels were prepared at two different energy levels (E1_s_ and E2_s_, see Table 2), varying *P_s_* and adjusting, consequently, the time of mixing according to Equation (2), and changing the fiber fraction in the aqueous phase. 

The composition of the samples was kept constant at 20% w/w of oil phase and 80% w/w of aqueous phase at three different fiber fractions (0.01, 0.02 and 0.03 w/w), see Table 2. The notation Ej.x.y.z used in sample codification reports the adopted energy level (j), the adopted power of mixing level (x), the fiber weight percentage (y) and the oil weight percentage (z). For example, E1.1.1.20 indicates the emulgel prepared at energy level E1_s_, power P1_s_, at 1% w/w of fiber and 20% w/w of oil. Emulgels E1.x.1.20 and E1.x.2.20 (i.e., all samples produced with the lowest specific energy of mixing, E1_s_*,* either 1% w/w or 2% w/w of fiber and 20% w/w of oil) showed instability after 24 h of preparation, independent of the specific power of mixing adopted, and it was not possible to proceed with rheological characterization. 

With the aim of investigating the effects of both oil and fiber fraction on the rheological properties of the emulgels, two further sets of samples were prepared, keeping constant the operating conditions and changing either the fiber fraction in the aqueous phase or the oil content. The specifications of the samples, codified as either E2.3.y.20 (samples at different fiber fractions) or E2.3.3.z (samples at different oil fractions), are listed in Table 3. 

When the oil content was changed, the fiber composition was kept constant at 3% w/w, based on the aqueous phase, whereas the oil weight fraction ranged from 10% up to 40%. All the emulgels were prepared while keeping the specific energy and power of mixing constant at 45.2 J⁄g and 0.151 W/g, respectively. These values of fiber fraction, energy and power were chosen according to the results of the isoenergy tests described in the Results and Discussion section.

### 2.3. Rheological Characterization 

All samples (both single phases and emulgels) were characterized with a controlled stress rheometer (Haake MARS III, Thermo Fisher Scientific, Germany) equipped with parallel plates geometry (diameter: 50 or 20 mm, and gap: 1.1 ± 0.1 mm or 2.0 ± 0.2 mm according to the samples’ consistency) and a Peltier system for temperature control. All samples were stored for 24 h at 4 °C before carrying out the characterization.

Frequency sweep tests at 25 °C and in linear conditions (preliminary stress sweep tests were carried out to evaluate the linear viscoelastic region) were performed between 0.1 and 10 Hz. All measurements were repeated three times; the results are shown in terms of mean value and standard deviation.

### 2.4. Microscopy Analysis

A contrast phase optical microscope (MX5300H, MEIJI, Japan, magnification 20× for all samples) and confocal microscope (TCS-SP8 based on an upright microscope DM6000, Leica Microsystems GmbH, Wetzlar, Germany, magnification 25×) were used to observe the microstructure of selected emulgels at room temperature. Microscopy was used mainly as a support for rheological investigation and as a tool to determine a physical property required in the model (i.e., the particle radius). Consequently, micrographs were obtained only for few selected samples considered representative of the common behavior. For the confocal microscopy investigation, no dyes were used to highlight the phases and the detection window was centered at 633 nm. The images were acquired using a scan speed of 400 Hz and a resolution of 1024 × 1024 pixels and the images were processed using LAS AF Lite software. Particle size distribution (PSD) of emulgels was measured with the contrast phase optical microscope; to avoid droplet aggregates, samples were diluted in distilled water with a weight ratio of 1:20 for emulgels with 20% w/w of oil and 1:30 for emulgels with 30% w/w of oil and mixed slowly using a magnetic stirrer to avoid modifications of the droplet distribution. A drop of diluted emulgel was placed on the microscope slide and observed; image analysis was carried out using an integrated micro-camera (Dhs Micro-cam 3.1 (Germany)). The PSD was obtained with a statistical analysis of data taken from the image processing software Dhs Particle Analysis (dhs image database, Germany), in which particles were colored to calculate their equivalent circular area diameter. PSD was modelled by a log-normal distribution, usually adopted for emulsions prepared with rotor-stator devices [35], in which the log-normal probability density function is:

(3) $$f(d)=\frac{1}{d\cdot \sigma_{\ln}\cdot \sqrt{2\pi }}\exp\left[\frac{-\left(\ln(d)-d_{\ln}\right)}{2\sigma_{\ln}^{2}}\right]$$

where $\sigma_{ln}$ is the standard deviation (µm) and $d_{ln}$ (µm) is the geometrical mean for the normal distribution of ln(*d*); the mean (*d*_s_) and the standard deviation ($\sigma_{s}^{2}$) of the log-normal distribution can be calculated from Equations (4) and (5), respectively:

(4) $$d_{s}=e^{d_{\ln}+\sigma_{\ln}^{2}/2}$$

(5) $$\sigma_{s}^{2}=e^{2d_{\ln}+\sigma_{\ln}^{2}}(e^{\sigma_{\ln}^{2}}-1)$$

### 2.5. Interfacial Characterization

To evaluate the potential contribution of interfacial properties to emulgel characteristics, interfacial tension measurements were carried out at the oil/water (O/W) interface by using an automated pendant drop tensiometer (FTA200, First Ten Angstroms, Portsmouth, VA, USA), based on Axisymmetric Drop Shape Analysis (ADSA) and equipped with fta32 v2.0 software. The instrument allows the formation of a pendant drop of one phase in the other one, using a syringe driven by an automated pump controlled by the software. An automated image viewing and capturing system was used and regression of the interfacial tension was performed directly with the instrument software by fitting the Bashforth–Adams equation to the drop shape. More details about the apparatus and the adopted procedure are available in the work of Biresaw et al. [36]. It is worth noticing that concentrations of additives used for emulgel preparation (i.e., fiber fraction 3% w/w in water and lecithin fraction 15% w/w in oil) were too high to allow for interfacial characterization under the same conditions using the pendant drop tensiometer. Indeed, at high lecithin concentrations, the system was too turbid and optical interferences could have affected the measurement; on the other hand, high fiber concentrations made the system too structured, hindering the flow in the syringe and the drop formation. Nevertheless, generally, when saturation conditions at the interface are obtained, no further changes in interfacial tension occur, even if bulk concentration is increased. Therefore, systems with low concentrations of fiber and lecithin were investigated while aiming at finding the saturation conditions and at understanding the potential effects of fiber at the interface. Pure O/W systems enriched with lecithin were studied up to the interface saturation condition (the so-called critical micellar concentration, CMC). At CMC, the effect of the fiber addition on the interfacial tension of the system was evaluated. Therefore, interfacial tension at saturation was used as a reasonable estimate of the value at concentrations adopted for emulgel production.

The oil phase was prepared at different concentrations of lecithin (in the range between 0.01 and 2% w/w), while the fiber was added in the range between 0.5 and 1.5% w/w into the pure water. Samples were prepared using the same procedures already descripted in Section 2.1. All tested samples are shown in Table 4. All the measurements were performed at room temperature (22 ± 1 °C), placing the aqueous solutions in a 100 mL glass Hamilton syringe (1710TLL) equipped with a stainless-steel needle (D = 20 gauge), and forming the drops in the oil phase inside a quartz cuvette (5 mL). After drop formation inside the oil phase, the drop profile was monitored at a constant volume for a maximum of 180 min and a quasi-equilibrium value (γ) was assumed when a plateau value was reached [37,38,39]. At a high concentration of lecithin, the test time was shorter than 180 min because the interfacial tension decreased quickly, and the drop detached from the needle. The tests were repeated two times and the results shown are the averaged values. 

### 2.6. Rheological Modelling

The rheological properties of both fiber suspensions and emulgels were modelled. According to Lupi et al. [40], dietary fiber suspensions behave as weak gels, wherein the network is based on aggregates built by the particles interacting with each other; therefore, they can be considered as particle gels, and they can be described as fractal systems [41]. Depending on the strength of the links between the flocs that build the fractal structure in comparison to that within the flocs themselves, two different behaviors can be observed: strong-link regime and weak-link regime [42]. In the present case, a weak-link regime was observed, as suggested by the increase in the critical strain value (evidencing the transition from linear to non-linear viscoelastic behavior in amplitude sweep tests) with fiber concentration [42] (data not shown). Therefore, the following fractal model can be proposed to relate the storage modulus of the dietary fiber suspensions to the particles’ volume fraction *ϕ* (v/v):

(6) $$G^{\prime }=\lambda \phi^{\frac{1}{3-D}}$$

where *λ* is a constant (in Pa) proportional to the interaction between primary particles, which form the fractal element, and to the inter-microstructural distance, and inversely proportional to the square of the diameter of the fractal flocs and to the diameter of the primary particles [43]. Parameter *D* is the fractal dimension and can be considered as a measure of the ”order” of microstructure [44] but also a measure of the density of the aggregated particles [45]; therefore, *D* can be considered as a measure of the structuration degree [46].

Assuming a proportionality between the volume fractions of fiber and the mass fractions *x_f_*, taken into account throughout parameter *λ* (in Pa), the fractal model can be rewritten as reported in Equation (7),

(7) $$G^{\prime }=\lambda^{\prime }x_{f}^{\frac{1}{3-D}}$$

where *λ*’ is a constant with similar meaning to *λ*. On the basis of the experimental evidence of a power law trend of dynamic moduli with frequency and considering that these suspensions behave as weak gels, as already said, the storage modulus *G*’ can be described by the power law:

(8) $$G^{\prime }\left(\omega \right)=k\cdot \omega^{n}$$

where *n* (dimensionless) and *k* (in Pa) are the fitting parameters. For these systems, the equation proposed by Muthukumar [47] can be used to relate the slope of the moduli, *n*, to the fractal dimension *D* (dimensionless):

(9) $$n=\frac{d\cdot \left(d+2-2D\right)}{2\cdot \left(d+2-D\right)}$$

where d represents the spatial dimensions (*d* = 3). For each frequency sweep test, the fractal dimension was calculated as in Equation (10):

(10) $$G^{\prime }\left(\omega \right)=k\cdot \omega^{\frac{3\cdot \left(5-2D\right)}{2\cdot \left(5-D\right)}}$$

The average values of fractal dimensions, calculated for the samples listed in Table 1, were used to fit experimental data of storage modulus versus fiber fraction (Equation (7)).

In the case of rheological modelling of emulgels, Palierne’s model for biphasic systems was considered as starting point. The elastic modulus of simple diluted monodisperse emulsions can be described as [48]:

(11) $$G_{r}=1+5H\phi$$

where *ϕ* is the oil volume fraction, *G_r_* is the relative shear elastic modulus (dimensionless) defined as the ratio between the modulus of the composite system and the modulus of the continuous phase:

(12) $$G_{r}=\frac{G}{G_{c}}$$

*H* is a parameter given by:

(13) $$H=\frac{\left(M-1\right)\left(19M+16\right)+\left(\frac{4\gamma }{RG_{c}}\right)\left(5M+2\right)}{\left(2M+3\right)\left(19M+16\right)+\left(\frac{40\gamma }{RG_{c}}\right)\left(M+1\right)}$$

with γ being the interfacial tension (in N/m), *R* is the droplet radius (in m) and *M* is the ratio between moduli (in Pa) of dispersed (*G_d_*) and continuous phases (fiber suspension in the present case, *G_c_*):

(14) $$M=\frac{G_{d}}{G_{c}}$$

For moderately concentrated systems (where hydrodynamic interactions cannot be neglected even if droplets are not closely packed) to take into account the interactions between particles, Palierne extended Equation (11) to undiluted systems [48]:

(15) $$G_{r}=\frac{1+3H\phi }{1-2H\phi }$$

If the interfacial tension term can be neglected because it is less important than the contribution of the bulk rheology, as is what happens when a rigid filler is present, Equation (13) becomes:

(16) $$H=\frac{\left(M-1\right)}{\left(2M+3\right)}$$

In addition, if the dispersed phase is less structured than the continuous one, for instance, when the dispersed phase is a viscous liquid, M << 1 and, therefore:

(17) $$H=-\frac{1}{3}$$

Equation (15) does not consider the crowding effects of the dispersing phase at high volume fractions; therefore, a modification of it was proposed, introducing, in the denominator, an “effective volume fraction” given by the product of a “crowding factor” *ψ* and *ϕ* [33] and yielding the Kerner–Lewis and Nielsen’s model:

(18) $$G_{r}=\frac{1+3H\phi }{1-2H\psi \phi }$$

A further modification introduces the crowding factor into the numerator, giving an extended version of the Kerner equation [49]:

(19) $$G_{r}=\frac{1+3H\psi \phi }{1-2H\psi \phi }$$

Equation (19) was also used in the literature, using different forms of the effective volume fraction, to model the behavior of some type of emulgels or filled gels based on protein gels, mainly with *M* > 1 and moduli increasing with volume fraction of the dispersed phase [14,15,16,28].

## 3. Results and Discussion

### 3.1. Rheological Characterization of Raw Phases

**Figure 1:**
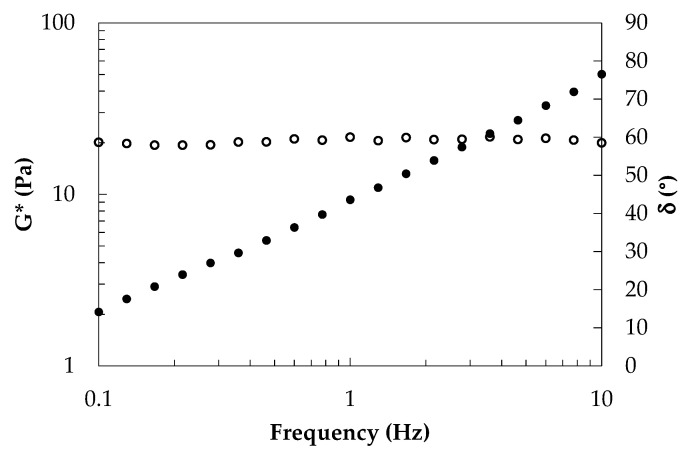
Complex modulus (full symbols) and phase angle (open symbols) of oil phase at 25 °C as frequency function.

The rheological behavior of the oil phase was investigated to evaluate the potential effects of lecithin addition on oil characteristics. It is worth noticing that, according to the literature [50], lecithin is an emulsifier not able to structure oils if polar solvents or co-surfactants are not present; therefore, no gelation should be expected. Experimental results (Figure 1) evidenced phase angle values greater than 45°, confirming that adopted oil phase is a viscoelastic liquid with a prevalent liquid-like behavior.

**Figure 2:**
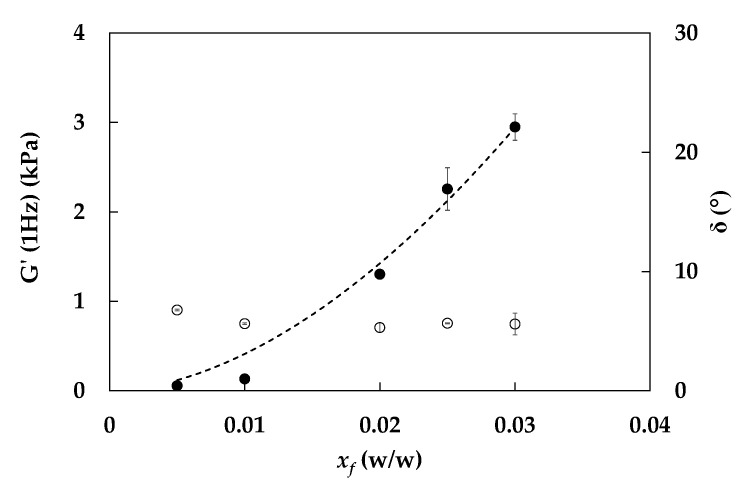
Storage modulus *G*’ (1 Hz, full symbols) and phase angle δ (°, open symbols) of fiber suspensions as a function of fiber fraction. Experimental data (full symbols) and fractal model fitting of *G*’ (dashed line, Equation (4)).

The rheological properties of O/W emulgels are related to aqueous phase characteristics, as already stated. Therefore, as a preliminary step to emulgel investigation, a rheological characterization of aqueous suspensions was carried out. In Figure 2, storage modulus *G*’ and phase angle δ at 1 Hz, obtained from frequency sweep tests, are shown as a function of fiber content; the average data are listed in Table 1. 

As shown, *G*’ increases non-linearly with fiber fraction, suggesting a strengthening of the network constituted by interacting fiber particles and aggregates with increasing fiber fraction. On the contrary, even if a slight difference is highlighted by statistical analysis between phase angles of samples prepared with 0.5% w/w of fiber and all other samples, this parameter seems to not be hugely affected by fiber concentration for percentages higher than 0.5% w/w (Table 1). Therefore, it can be concluded that structuration degree of samples is constant with fiber fraction starting from 1% w/w, with an average value of 5.6° ± 0.2°. 

The fractal model in Equation (7) was adopted to fit experimental data, as already performed for similar systems in a previous work [34]. The fractal dimensions *D* were calculated from the frequency sweep tests using Equation (10) (Table 1). From the statistical analysis, it is possible to observe that the fractal dimension of suspensions is independent of fiber fraction for samples produced with *x_f_* > 0.01; an average value of 2.44 ± 0.01 (−) was measured and used to fit the experimental data of *G*’ vs. *x_f_* with Equation (7), obtaining *λ*’ = (15.6 ± 0.7)∙10^5^ Pa. It is worth noticing that, using a fiber sample different than that investigated in the previous work, both parameters differ than those previously obtained [34], even if differences are more relevant for *λ*’ than for *D*.

### 3.2. Rheological Characterization of Emulgels: Effect of Process and Fiber Concentrations

According to results of frequency sweep tests, all studied emulgels exhibit a similar behavior of moduli with frequency of oscillation (ω): both moduli are almost linear in a log–log scale with *G*’ almost one order of magnitude greater than *G*″.

**Figure 3:**
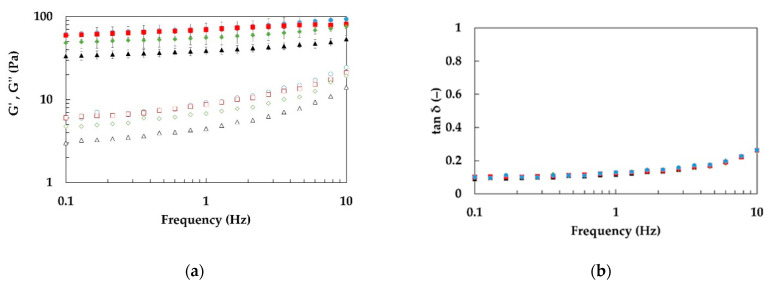
Frequency sweep tests of emulgels (1% w/w dietary fiber, 20% w/w oil phase) at different specific mixing powers and at specific energy E2_s_ = 45.2 J/g. (**a**) Storage modulus (*G*’, solid symbols) and loss modulus (*G*″, open symbols); (**b**) loss tangent (tanδ, solid symbols). E2.1.1.20: black triangles, E2.2.1.20: green diamonds, E2.3.1.20: red squares, E2.4.1.20: blue circles.

**Figure 4:**
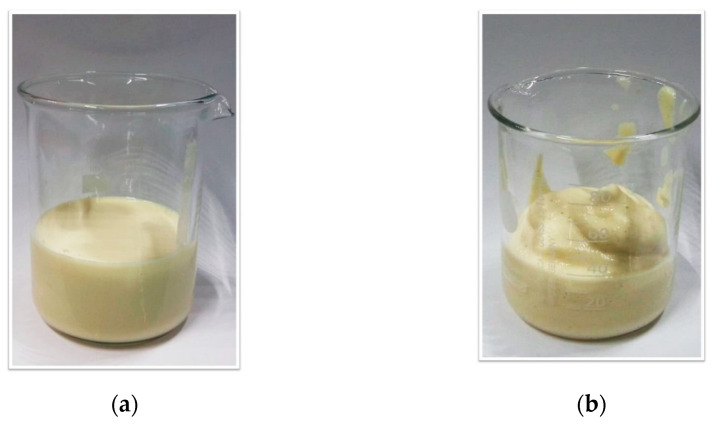
Visual aspect of samples E2.3.1.20 (**a**) and E2.3.3.20 (**b**).

In Figure 3, the trend of *G*’, *G*″ (a) and the loss tangent tan(δ) (b) versus ω is shown for samples of the series E2.x.1.20, taken as an example, increasing the power of mixing. This behavior is typical of weakly structured materials [51]. This result was already observed for O/W emulsions structured with citrus fibers [52] and this weak gel behavior is confirmed by the visual inspection of samples (as an example, the visual aspect of samples E2.3.1.20 and E2.3.3.20 is shown in Figure 4).

**Figure 5:**
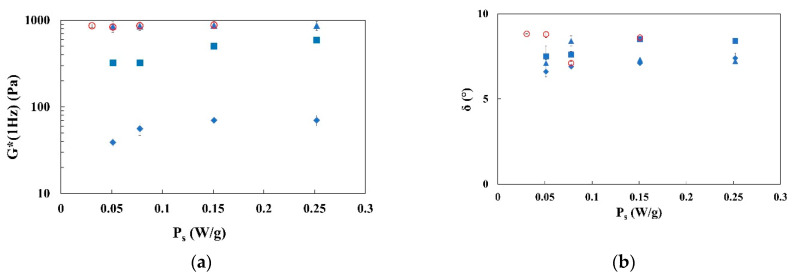
(**a**) Complex modulus G* (1 Hz) and (**b**) phase angle δ (1 Hz) of emulgels at different fiber concentrations for different specific powers of mixing (P_s_) at the two energy levels E1_s_ = 22.6 J/g (open symbols) and E2_s_ = 45.2 J/g (solid symbols). E2.x.1.20: blue diamonds, E2.x.2.20: blue squares, E2.x.3.20: blue triangles, E1.x.3.20: red circles.

Complex modulus (G*) and phase angle (δ) at 1 Hz for all investigated samples are shown in Figure 5 and listed in Table 2. Looking at the results in Figure 5a, complex modulus (G*) increases with the fiber content and, with the highest fractions of fiber added to water; no differences are visible among data, neither with energy of mixing nor with power.

It is worth noting that for emulgels E2.x.1.20 (i.e., samples with the lowest fiber amount), the complex modulus increases with *P_s_* up to a power of mixing lower than approximately 0.15 W/g; after that, an apparent plateau value of G* is reached, as already found by Lupi et al. [40] for citrus fiber suspensions. The trend is similar for emulgels E2.x.2.20, in which the complex modulus increases slightly up to the power value of 0.15 W/g and remains constant when power of mixing is increased further. On the contrary, for samples produced with 3% w/w of fiber (i.e., E1.x.3.20 and E2.x.3.20), the complex modulus becomes independent of the specific power of mixing and energy level adopted.

In Figure 5b, the phase angle values are shown; their inverse value can be related to the degree of structuration of samples [51]. According to the figure and to the statistical analysis (Table 2), samples E1.x.3.20 show a phase angle higher than other samples, confirming that at the lowest adopted energy, the samples are less structured with respect to other emulgels. Furthermore, samples E2.x.2.20 show a slight decrease in structuration degree when increasing the power of mixing; this can be related to the shorter mixing time adopted to disperse these consistent samples.

The fiber suspensions data were related to those of emulgels obtained in the same conditions. The ratio between the complex moduli of emulgels G* and the fiber suspensions (i.e., the continuous phase) G_c_* was calculated at 1 Hz, according to Equation (12):

(20) $$G_{r}^{*}\left(1\mathrm{Hz}\right)=\frac{G^{*}\left(1\mathrm{Hz}\right)}{G_{c}^{*}\left(1\mathrm{Hz}\right)}$$

**Figure 6:**
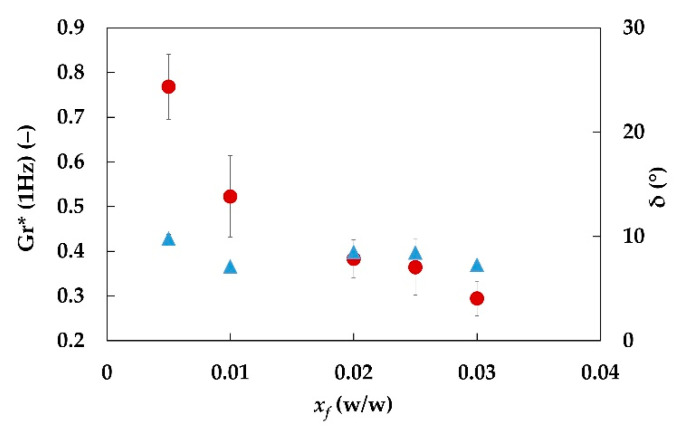
Relative complex modulus $G_{r}^{*}$ (1 Hz) (red circles) and phase angle δ (1 Hz) (blue triangles) of emulgels with 20% w/w of oil phase, at different fiber fraction and constant preparation conditions (E2.3.y.20 samples). Operating energy and power of mixing for emulgels and aqueous suspensions: *E_s_* = 45.2 J/g and *P_s_* = 0.151 W/g.

The trend of $G_{r}^{*}$ as a function of fiber fraction is shown in Figure 6. A monotonous decrease is observed. Indeed, even if both suspensions and emulgels show an increase in moduli with increasing fiber content, the complex modulus of hydrogel increases more than the complex modulus of the corresponding emulgel.

### 3.3. Microstructural Analysis of Emulgels

**Figure 7:**
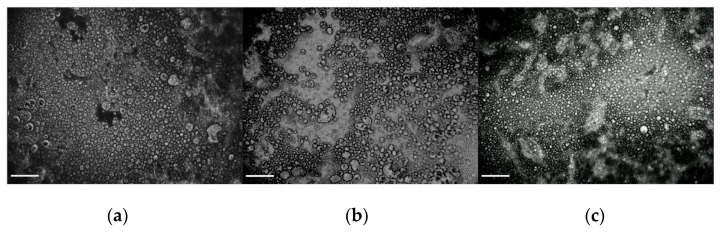
Contrast phase micrographs for samples E2.3.1.20 (**a**), E2.3.3.20 (**b**) and E2.3.3.30 (**c**); magnification 20×; reference bar corresponds to 100 µm.

The microstructure of emulgels was investigated, from a morphological point of view, with optical and confocal microscopy. The micrographs reported in Figure 7, obtained with a contrast phase microscope, show the microstructure of samples E2.3.1.20, E2.3.3.20 and E2.3.3.30 prepared with the same operating conditions, but varying compositions. The mean diameter (Equation (4)) and the polydispersity (standard deviation of log-normal distribution, Equation (5)) of samples E2.3.1.20, E2.3.3.20 and E2.3.3.30 were obtained and are reported in Table 5. According to the statistical analysis carried out by the ANOVA test (*p* < 0.05) reported in Table 5, there are no differences among mean diameters of the dispersed phase when either fiber content in the aqueous phase or oil content in the emulgel is varied, even if polydispersity (which seems to be quite broad), measured by standard deviation values, is slightly different for the three samples.

**Figure 8:**
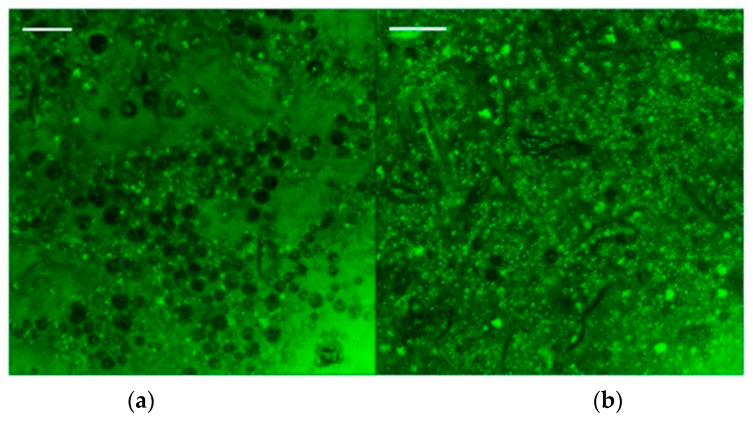
Confocal micrographs for samples E2.3.1.20 (**a**), E2.3.3.20 (**b**); magnification 25×; reference bar corresponds to 50 µm.

The partitioning of the two phases becomes more evident with confocal microscopy as shown in Figure 8. A dispersed oil phase (black) and a continuous fluorescent matrix composed of a fiber network (green) are detected.

As expected, the emulgel with the highest fiber fraction (Figure 8b) shows a more compact network in which many fluorescent particles are present, whereas the droplets seem to be less visible, i.e., more wrapped by the surrounding matrix of hydrated fibers. The figures suggest that, mostly at the highest fiber fraction, the oil droplets are not perfectly spherical, probably owing to the break-up action of the mechanical device.

At lower fiber fractions (Figure 8a), a poorly compacted network and some droplet clustering can be observed. Moreover, it is possible to observe that for a fiber fraction of 1% w/w, the network formed is significantly less dense (less colored) than the one formed with 3% w/w, confirming the strengthening effect of fiber in the aqueous phase found in the rheological tests. Figure 8 shows, in both cases, a network formed by fiber aggregates surrounding the oil droplets with particles (green spots) close to droplets. As reported previously by other authors [52,53], this interposition of the particles between the two phases can further improve the stability of the emulgels provided by the fiber network.

### 3.4. Interfacial Properties

Interfacial tension values of oil containing lecithin, at the interface with pure water, are listed in Table 4 as a function of lecithin content. Increasing the concentration, interfacial tension decreases significantly down to an almost constant value, corresponding to the saturation conditions (CMC), observed for lecithin fractions greater than 0.5% w/w.

The effects of fiber (1% w/w) were investigated at the interface with pure oil to understand the potential interfacial characteristics of the pure fiber. A slight reduction with respect to the unloaded interfaces is observed even if it is much lower than that caused by lecithin at low concentrations (0.01% w/w).

At the CMC, different amounts of fiber were added to investigate its effect on interfacial tension and the potential interactions with lecithin. It is possible to observe (Table 4) that the interfacial tension of the system is almost constant. In particular, at the lowest investigated fiber concentration (0.5% w/w), the measured tension is slightly higher (approximately 21%) than that obtained when using pure water, whereas further increases in fiber fraction (up to the largest investigable value) yield the same tension of the interface with pure water.

Even though other data on simultaneous adsorption of fiber and surfactants at the interface are not available, the obtained results could be analyzed in light of works in the literature about systems where synergistic and/or competitive adsorption of components from two different phases was investigated. For instance, Fainerman et al. [54] studied the adsorption of surfactants (in water phase) and alkanes (in oil phase) at water/oil interfaces and they observed that with increasing surfactant concentration, the alkane adsorption at the interface goes through a maximum and becomes almost zero when a large amount of surfactant is present. At intermediate concentrations, a cooperative effect with a reciprocal attraction at the interface was observed, whereas at higher concentrations, the surfactant seemed to be able to replace the other compound (competitive effect), controlling the measured interfacial tension. Even though the investigated systems are far from those studied in this work, these results seem to suggest that the simultaneous adsorption of different species from different phases can be competitive or cooperative, depending on their behavior and relative amount. Starting from this evidence within the literature, it could be speculated that the presence of fiber at the interface could hinder lecithin adsorption for a competitive effect, yielding, as a result, a higher tension value with respect to the lecithin alone. As the fiber concentration increases, this effect is no longer evident; it could be supposed that at higher fiber concentrations, the increase in gelling effect could limit fiber migration to the interface, reducing the competitive effects and promoting lecithin adsorption. Nevertheless, further investigation outside the aim of the present work would be necessary to understand the phenomena involved at the interface.

Starting from the obtained data, it can be concluded that for lecithin fractions greater than 0.5% w/w and for fiber fractions greater than 1% w/w, an apparent plateau in interfacial tension value is obtained and it is equal to 1.40 ± 0.08 mN/m, computed as the average of all values obtained at investigated plateau conditions. According to the obtained results, it seems that this value can be assumed as a reasonable estimate of interfacial tension in investigated emulgels, even if larger fractions of lecithin and fiber are used in these systems.

### 3.5. Rheological Characterization and Modelling of Emulgels

**Figure 9:**
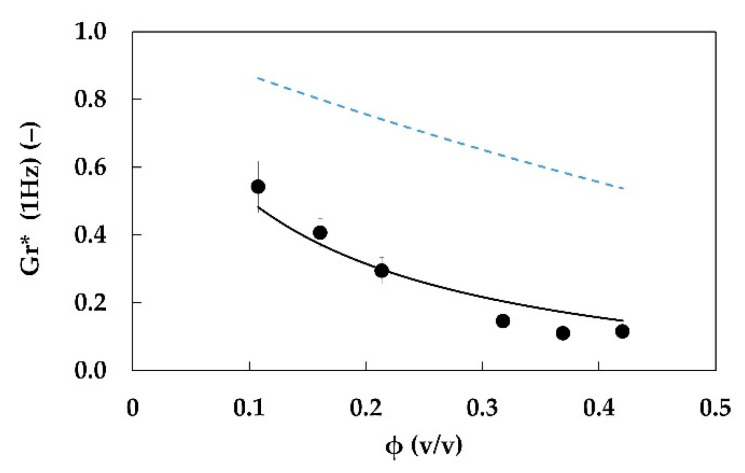
Relative complex modulus of emulgels as a function of volumetric fraction *ϕ*; experimental data (black circles) are compared with the Palierne model (Equation (15) (dashed blue line)) and a modified Palierne model (Equation (18) (solid black line)).

The volume fraction of the dispersed phase in emulgels was varied, keeping the process conditions (at E2_s_ = 45.2 J/g and *P_s_* = 0.151 W/g corresponding to plateau conditions) and the concentration of fiber in the aqueous phase (3% w/w) constant. The results obtained from the rheological characterization, in terms of the complex modulus and phase angle at 1 Hz, are shown in Table 3. According to the data, the increase in the oil fraction added to emulgels results in a decrease in the complex modulus, as shown in Figure 9; this behavior is known and expected for emulgels of non-interacting particles as described by Dickinson et al. [55] and Lupi et al. [7]. On the other hand, phase angles do not seem to be dependent on the oil phase content and no statistically significant difference is observed among obtained values.

The experimental values of relative complex moduli of samples at 1 Hz were compared to results obtainable using the Palierne model (Equation (15)) estimating the bulk and interfacial rheological contribution of parameter *H* (Equation (13)). Particularly, the droplet radius R was assumed as the mean value of experimental data obtained with optical microscopy for emulgels with 3% of fiber fraction (see Table 5) (R= 3.0 ± 0.2 µm), M was computed with G* at 1 Hz of single phases (M = 3.7·10^−3^) and *H* = −0.270 was obtained; this value evidences that, in the present case, neglecting the interfacial contribution could yield a relevant error in *H* estimates.

The comparison between experimental data and Palierne’s model is shown in Figure 9 and highlights that the model is unable to describe the experimental results, probably owing to the deviations of investigated systems from the theoretical assumptions. In fact, the Palierne model (Equation (15)) was proposed for systems with monodisperse non-interacting and non-deformable spherical particles, uniformly distributed within the continuous phase; furthermore, in the Palierne model, both phases are supposed to be elastic liquids. Fiber emulgels are quite different from systems described by Palierne: they do not show uniform distribution, but irregular particles with a coarse distribution are involved, owing to the intense mechanical action given by the rotor–stator device. Furthermore, particles are deformable and interactions among them are possible; finally, approximations in determination of interfacial contribution could also affect the final result.

Because of these deviations, a modified form of the Palierne model can be proposed to fit experimental data, and Equation (18) can be used to describe the observed behavior even if “*ψ*”, in this case, is mainly an empirical parameter taking into account all deviations from ideal Palierne behavior more than high volume conditions. If Equation (18) is adopted to fit experimental data, a good agreement can be observed even if larger deviations are observed at higher volume fractions. The obtained fitting parameter is *ψ* = 15 ± 2 (−).

With the aim of evaluating the potential ability of the proposed model to describe emulgels with different compositions, a comparison was carried out between model forecasts and experimental data obtained for emulgels prepared using the same specific energy and power and different fiber fractions, i.e., samples E2.3.y.20. It can be seen (Table 6) that, at low fiber concentrations, parameter M approaches unity because the complex modulus of the water phase is close to that of oil phase. In these conditions, the interfacial contributions seem to play a relevant role in parameter estimation, owing to the low structuration level of both phases.

With increasing fiber fraction, the modulus of the dispersing phase increases and, therefore, the relevance of interfacial term decreases, even if *H* is quite different with respect to the value expected for situations where it can be neglected (i.e., *H* = −1/3, see Equation (17)). When experimental data and model results are compared, for *x_f_* > 0.01, differences are very low, whereas at low *x_f_*, the model seems to not be working. This result also confirms microscopy observations: as already remarked, samples produced at low fiber concentrations possess an apparent “loose” structure with a less dense (and colored) network.

It could be speculated that, at low fiber concentrations, interfacial phenomena become prevalent (with respect to the bulk ones) and a more accurate estimate of droplet radius and interfacial tension could improve the results. Nevertheless, other phenomena could be involved and further investigation on this issue is necessary.

However, the proposed model, containing only one fitting parameter and based only on physical properties, seems to be able to describe the rheological behavior of emulgels with fiber fractions between 0.2 and 0.3 and oil fractions up to 0.4 very well, and it could be used to design systems with desired properties within these ranges of composition.

## 4. Conclusions

Emulgels produced by structuring an aqueous phase with dietary fiber were prepared with a rotor–stator device. The preliminary investigation of single phases demonstrated that the oil phase is a viscoelastic liquid with a liquid-like behavior, whereas the water phase behaves in a similar manner to a weak gel structured by dietary fiber. Aqueous gelled suspensions were also characterized by varying fiber concentrations and a fractal model was considered suitable to fit the experimental data.

Then, the effect of operating conditions on the rheological properties of emulgels was studied. It was observed that the specific power and energy of mixing influence the consistency and the stability of the emulgels produced at low fiber concentrations, whereas, at higher concentrations, the strength of interactions does not change with mixing conditions (i.e., with both power and energy of mixing). On the other hand, the network extension, described by the phase angle, seems to always be independent of the operating conditions.

Microstructural investigation showed that the increase in fiber concentration leads to an improvement in the stability of emulsions, probably thanks to the reduction in the dispersed phase droplets’ dimension and to the increase in viscosity of the continuous phase.

The rheological modelling of emulgels was also carried out. Palierne model was not able to interpret the experimental results, owing to the deviation of the emulgels’ characteristics from the ”ideal” system described by Palierne. An empirical parameter was used to modify the Palierne model and to fit the experimental data.

These results show that the process conditions and the fiber concentrations together with the ratio between the dispersed and the continuous phase can be varied according to the expected rheological properties of the final products. This work can be useful for designing new functional foods using dietary fiber, such as dressings, creams or structured food emulsions in general.

## Figures and Tables

**Table 1:** Aqueous phases obtained by increasing fiber concentration; mixing conditions fixed at *E_s_* = 45.2 J/g and *P_s_* = 0.151 W/g. G* is the complex modulus, δ is the phase angle and *D* is the fractal dimension (Equation (10)). Different letters, for the same parameter, refer to significantly different values.

ID	*P_s_*(W/g)	Ω (rpm)	*t* (s)	Fiber Fraction (w/w)	G* (1 Hz) (Pa)	δ (1 Hz) (°)	*D* (−)
H_0.5	0.151	8000	300	0.005	57 ± 2 a	6.77 ± 0.06 a	2.42 ± 0.01 a
H_1				0.01	140 ±10 b	5.75 ± 0.09 b	2.444 ± 0.002 b
H_2				0.02	1310 ± 50 c	5.3 ± 0.3 b	2.453 ± 0.005 b
H_2.5				0.025	2300 ± 200 d	5.66 ± 0.05 b	2.438 ± 0.003 ab
H_3				0.03	3000 ± 100 e	5.57 ± 0.09 b	2.446 ± 0.007 b

**Table 2:** Samples prepared at two specific energy levels: E1_s_ = 22.6 J/g and E2_s_ = 45.2 J/g. At different speeds (Ω), specific powers of mixing (*P_s_*), mixing times (*t*) and fiber fractions (w/w). G* is the complex modulus and δ is the phase angle. ANOVA was performed on the set of samples having the same fiber concentration. Different letters, for the same parameter and for the set of samples having the same fiber concentration, refer to significantly different values.

Isoenergy E1_s_ = 22.6 J/g						
ID	*P_s_* (W/g)	Ω (rpm)	*t* (s)	Fiber Fraction (w/w)	G* (1 Hz) (Pa)	δ (1 Hz) (°)
E1.1.3.20	0.031	4000	738	0.03	860 ± 40 a	8.82 ± 0.02 a
E1.2.3.20	0.051	5000	442	0.03	840 ± 60 a	8.2 ± 0.2 a
E1.3.3.20	0.078	6000	290	0.03	860 ± 30 a	7.1 ± 0.1 b
E1.4.3.20	0.151	8000	150	0.03	880 ± 90 a	8.6 ± 0.3 a
**Isoenergy E2_s_ = 45.2 J/g**						
**ID**	***P_s_*** **(W/g)**	**Ω** **(rpm)**	***t*** **(s)**	**Fiber fraction** **(w/w)**	***G*** * (1 Hz) **(Pa)**	**δ (1 Hz)** **(°)**
E2.1.1.20	0.051	5000	886	0.01	39 ± 4 a	6.6 ± 0.3 a
E2.2.1.20	0.078	6000	580	0.01	56 ± 9 ab	6.9 ± 0.1 ab
E2.3.1.20	0.151	8000	300	0.01	70 ± 5 b	7.1 ± 0.1 ab
E2.4.1.20	0.252	10,000	179	0.01	70 ± 10 b	7.4 ± 0.3 b
E2.1.2.20	0.051	5000	886	0.02	323 ± 5 a	7.5 ± 0.6 a
E2.2.2.20	0.078	6000	580	0.02	323 ± 2 a	7.7 ± 0.2 ab
E2.3.2.20	0.151	8000	300	0.02	500 ± 30 b	8.51 ± 0.01 b
E2.4.2.20	0.252	10,000	179	0.02	590 ± 40 c	8.4 ± 0.1 ab
E2.1.3.20	0.051	5000	886	0.03	850 ± 120 a	7.1 ± 0.2 b
E2.2.3.20	0.078	6000	580	0.03	860 ± 90 a	8.4 ± 0.3 a
E2.3.3.20	0.151	8000	300	0.03	870 ± 70 a	7.3 ± 0.1 b
E2.4.3.20	0.252	10,000	179	0.03	860 ± 100 a	7.2 ± 0.1 b

**Table 3:** Emulgels produced by varying either the fiber fraction or the fraction of dispersed phase (oil); mixing conditions fixed at E2_s_ = 45.2 J/g and *P_s_* = 0.151 W/g. G* is the complex modulus and δ is the phase angle. Different letters, for the same parameter and for the same set of samples, refer to significantly different values.

ID	Fiber Fraction (w/w)	Oil Weight Fraction (% w/w)	Oil Volume Fraction (% v/v)	G* (1 Hz) (Pa)	δ (1 Hz) (°)
E2.3.3.10	0.03	10	10.8	1600 ± 100 a	7.6 ± 0.2 a
E2.3.3.15		15	16.1	1200 ± 60 b	7.9 ± 0.1 a
E2.3.3.20		20	21.4	870 ± 70 c	7.3 ± 0.1 a
E2.3.3.30		30	31.8	433 ± 30 d	7.6 ± 0.2 a
E2.3.3.35		35	36.9	327 ± 20 d	8.0 ± 0.3 a
E2.3.3.40		40	42.0	340 ± 20 d	7.3 ± 0.3 a
E2.3.0_5.20	0.005	20	21.4	43 ± 3 e	9.8 ± 0.4 e
E2.3.1.20	0.01			70 ± 5 e	7.1 ± 0.1 f
E2.3.2.20	0.02			501 ± 35 f	8.5 ± 0.1 g
E2.3.2_5.20	0.025			830 ± 50 g	8.4 ± 0.5 g
E2.3.3.20	0.3			870 ± 70 g	7.3 ± 0.1 f

**Table 4:** Samples investigated by interfacial test and interfacial tension values (γ). Lecithin was added to oil phase; fiber to water phase.

Lecithin Fraction (w/w)	Fiber Fraction (w/w)	γ (mN/m)
**-**	-	23.0 ± 0.1
-	0.01	16.0 ± 0.2
10^−9^	-	23.0 ± 0.1
0.0001	-	9.43 ± 0.07
0.001	-	3.9 ± 0.1
0.005	-	1.43 ± 0.05
0.005	0.005	1.72 ± 0.03
0.005	0.01	1.4 ± 0.1
0.005	0.015	1.40 ± 0.08
0.01	-	1.40 ± 0.07
0.02	-	1.49 ± 0.08

**Table 5:** Mean diameter d_s_ (Equation (4)) and standard deviation σ_s_ (Equation (5)) of the log-normal distribution. Different letters, for the same parameter, refer to significantly different values.

ID	d_s_ (μm)	σ_s_ (μm)
E2.3.1.20	7.1 ± 0.8 a	6 ± 1 a
E2.3.3.20	6.5 ± 0.3 a	4.3 ± 0.1 ab
E2.3.3.30	5.5 ± 0.6 a	3.1 ± 0.5 b

**Table 6:** Comparison between experimental (G_r_*_exp_) and model (G_r_*_mod_) values of the complex modulus for samples at different fiber contents (*x_f_*) prepared at *E_s_* = 45.2 J/g and *P_s_* = 0.151 W/g. Sample E2.3.3.20 was used in fitting parameter determination.

Sample ID	*x_f_* (‒)	G_r_*_exp_ (‒)	*M* (‒)	*H* (‒)	G_r_*_mod_ (‒)	Error (%)
E2.3.0_5.20	0.005	0.6 ± 0.1	0.19	0.190	<0	-
E2.3.1.20	0.01	0.52 ± 0.08	0.081	0.084	2.35	300%
E2.3.2.20	0.02	0.38 ± 0.03	0.0084	‒0.208	0.37	‒4%
E2.3.2_5.20	0.025	0.36 ± 0.05	0.0048	‒0.253	0.32	‒14%
E2.3.3.20	0.03	0.29 ± 0.04	0.0037	‒0.270	0.30	1%

## Data Availability

The data used to support the findings of this study can be made available by the corresponding author upon request.
